# Sven Ivar Seldinger (1921-1998): The Founding Father of Interventional Radiology

**DOI:** 10.7759/cureus.60397

**Published:** 2024-05-16

**Authors:** Hamza Hafiani, Rayhana Charif Saibari, Nasma Morsad, Amal Rami

**Affiliations:** 1 Radiology, Cheikh Khalifa International University Hospital, Mohammed VI University of Health Sciences, Casablanca, MAR; 2 Radiology, Cheikh Khalifa International University Hospital, Casablanca, MAR

**Keywords:** catheter, technique, interventional radiology, radiology, seldinger

## Abstract

Sven Ivar Seldinger, a Swedish radiologist, laid the cornerstone of modern interventional radiology in 1953 with the introduction of his innovative technique for catheter insertion. This technique, known as the Seldinger technique, represents a pivotal advancement in medical procedures, offering a safer, less invasive method for vascular access. The elegance and effectiveness of this technique not only refined angiography and other catheter-based interventions but also heralded the birth of interventional radiology as a major therapeutic specialty. This article delves into Seldinger's life, the genesis of his technique, its profound impact on medical practice, and his enduring legacy that continues to resonate across numerous medical specialties today. By pioneering minimally invasive approaches to treatment, Seldinger's innovation has significantly alleviated patient risk and discomfort, broadening the spectrum of therapeutic options available in modern medicine.

## Introduction and background

**Figure 1:**
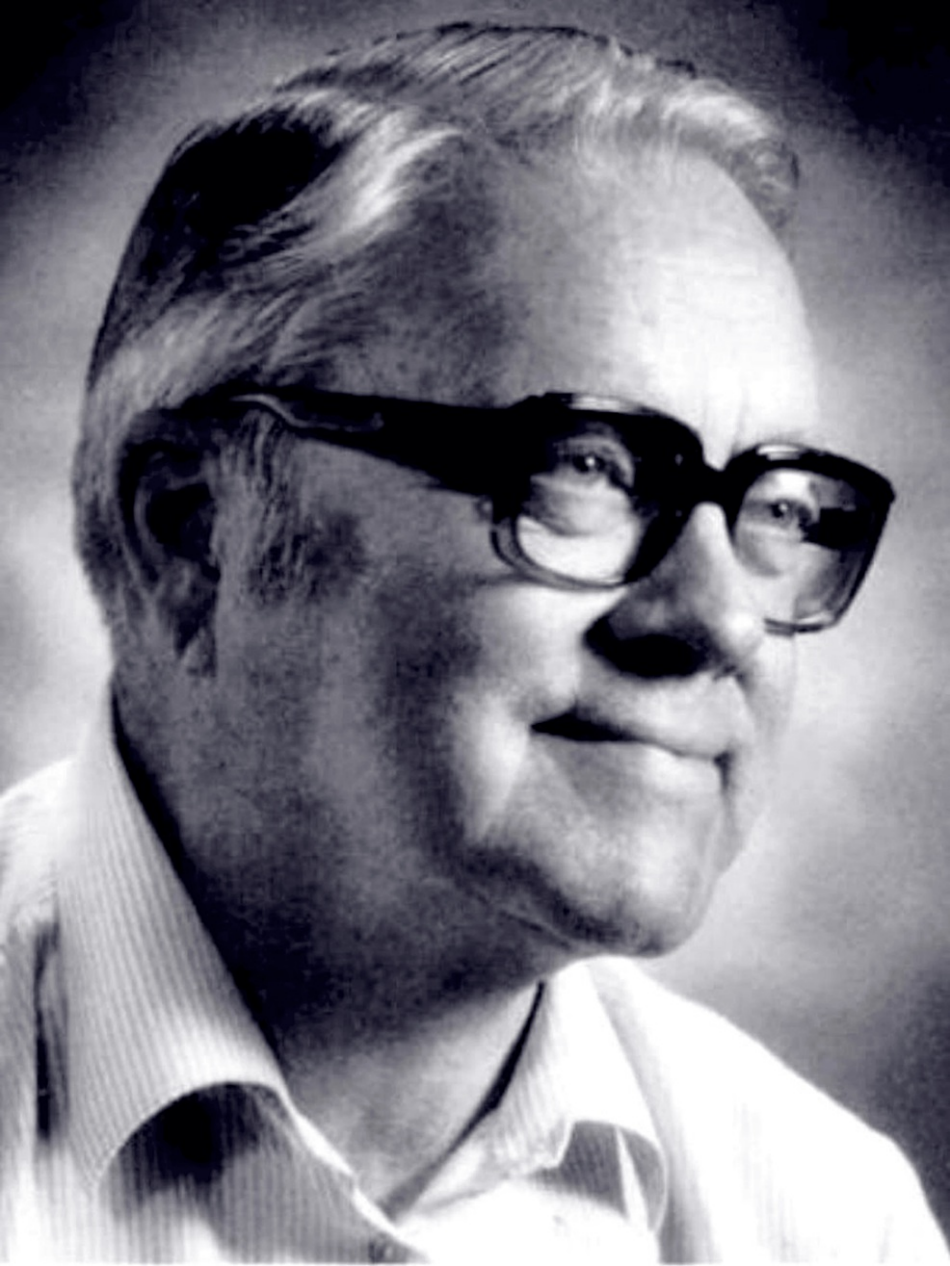
Dr. Sven Ivar Seldinger Source: [1]

The primary aim of this article is to explore the enduring legacy of Sven Ivar Seldinger (Figure 1), whose technique fundamentally altered the practice of catheterization. The article will cover his early life, the development of the Seldinger technique, its impact on medical procedures, and his lasting influence on the field of interventional radiology (IR).

## Review

Seldinger's early life and career

Born on April 19, 1921, in Mora, Dalecarlia, Sweden, Seldinger was influenced by a familial legacy of technical ingenuity associated with the Mora Mechanical Workshop. His early environment was steeped in technical creativity, which profoundly impacted his intellectual development [2].

Seldinger's academic journey began at the Karolinska Institute in 1940. His intelligence and independence marked him early on as a standout student. After completing his initial medical training in 1948, he delved deeply into radiology, driven by an innate curiosity and innovative spirit. He demonstrated his early ideas using phantom models, showing how catheter insertion through the femoral route could potentially reach all arteries in the human body [3].

The creation of the Seldinger technique marked a significant milestone in his career. As a young resident at the Karolinska Hospital, Seldinger conceived a less invasive method for aortography that involved using a flexible catheter introduced through a needle. This method significantly improved the safety and efficacy of angiographic procedures [2,3]​​.

Despite the groundbreaking nature of his technique, Seldinger initially encountered skepticism. His department head at Karolinska did not believe the technique warranted a doctoral thesis. Nonetheless, Seldinger persisted, and the eventual publication of his technique in "Acta Radiologica" in 1953 [3] helped his method gain recognition and acceptance first in Europe and subsequently in the United States. This recognition transformed the practice of radiology, facilitating angiography and interventional procedures worldwide, like central line insertion in anesthesiology​​​.

Seldinger continued his career at the Karolinska Institute until 1966, after which he returned to his hometown of Mora to lead the radiology department at the local hospital. His continued work and dedication significantly advanced the field of medical imaging and intervention, cementing his status as a pioneer whose innovations remain fundamental to medical practice today [4].

Development of the Seldinger technique

Sven Ivar Seldinger's development of his renowned technique marked a significant breakthrough in the field of radiology and interventional medicine. Inspired by earlier methods and pioneers in catheterization, Seldinger was notably influenced by the works of Pedro L. Fariñas [5], who in 1941 described an over-wire technique of catheter insertion. Fariñas's method, although a precursor to percutaneous approaches, involved more invasive and less flexible techniques.

While working at the Karolinska Institute in the early 1950s, Seldinger observed the limitations of these existing methods, which either required surgical exposure of vessels or were limited by the sizes of the catheters and needles used. He also innovated upon later techniques developed by André Frédéric Cournand [6] and others [7], who had similarly explored vascular access methods.

Seldinger's innovative method, which cleverly integrated a needle, a guidewire, and a catheter in a sequential approach, significantly reduced vascular trauma and enhanced the flexibility of catheter placement, initiating a transformative era in medical procedures.

The Seldinger Technique

**Figure 2:**
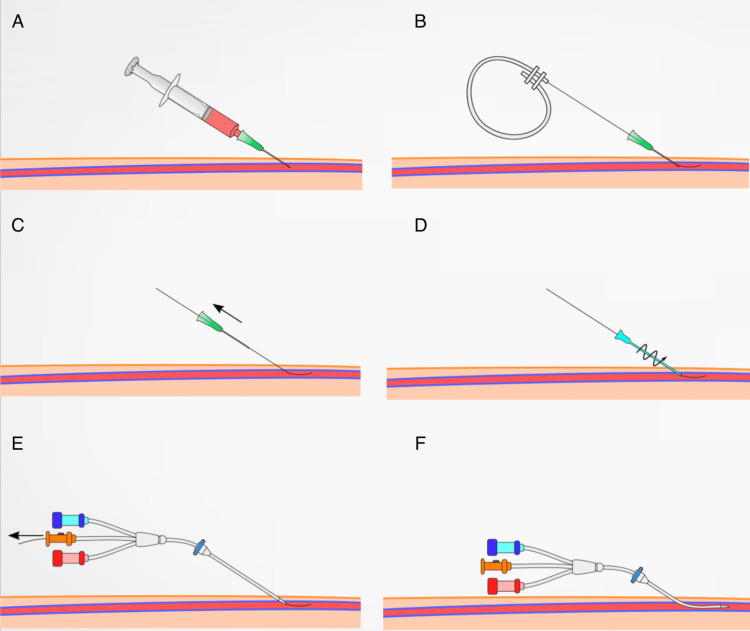
Different steps of the Seldinger technique. Images from Wikimedia Commons, the free media repository

The technique, developed in 1953, has streamlined numerous medical procedures by allowing safer and more effective catheter placement into blood vessels. It includes 6 sequential steps (Figure 2) beginning with percutaneous needle insertion where a sharp, hollow needle into the desired blood vessel. This step establishes an initial access route through the skin and into the vessel. Through the needle, a flexible, round-ended guidewire is introduced into the vessel. The flexibility of the guidewire helps navigate through the vascular system without causing significant trauma to the vessel walls. Once the guidewire is adequately positioned within the vessel, the needle is carefully withdrawn, leaving the guide-wire in place. This step reduces the trauma to the vessel as the more rigid needle is removed. A catheter is then threaded over the guidewire. The catheter can navigate along the guidewire to reach the specific area of interest within the vascular system. After the catheter is in the desired position, the guidewire is withdrawn, leaving the catheter in place for the intended intervention. Finally, the catheter is secured to ensure it remains in place during the procedure, and its position may be confirmed using imaging techniques like fluoroscopy to ensure accurate placement for the diagnostic or therapeutic process.

Impact on medical procedures

The Seldinger technique enabled a paradigm shift in interventional radiology(IR) by facilitating safer and more refined catheter-based interventions. Initially developed to improve the safety of catheter insertions, this technique quickly became foundational, greatly enhancing the scope and capabilities of angiography and other vascular interventions [8].

As detailed in historical accounts, the adoption of the Seldinger technique coincided with significant advancements in IR, shifting the field from a predominantly diagnostic discipline to a more dynamic therapeutic specialty. This technique allowed interventionalists to perform a wide range of minimally invasive procedures that were previously unthinkable, broadening the applications of IR across various organ systems and medical conditions​ [9]​.

The widespread integration of the Seldinger technique into clinical practice catalyzed the development of new devices and methods, enhancing the precision and safety of interventional radiology procedures. This included the introduction of specialized catheters and tools that enabled targeted treatments for complex conditions, thereby reducing the need for open surgeries and improving patient outcomes [10]​. The evolution of these techniques and tools contributed significantly to the emergence of IR as a distinct medical specialty, distinguishing it from traditional diagnostic radiology and positioning it as a vital therapeutic field [11].

Furthermore, the flexibility and reduced risk profile of the Seldinger technique fostered innovation in areas such as cancer treatment, where percutaneous methods became crucial in the management of tumors, particularly in organs like the liver and kidneys. The technique's adaptability facilitated the development of new therapeutic approaches, including embolization and localized drug delivery, which are now cornerstones in the treatment of various cancers​ [9].

In summary, the introduction and subsequent adoption of the Seldinger technique significantly expanded the capabilities of interventional radiology, transforming it from a niche area focused on diagnostic imaging to a broad, dynamically evolving field of medicine. This shift not only enhanced the therapeutic potential of IR but also established it as a critical component of modern healthcare, offering less invasive alternatives for a wide range of medical interventions [11].

Seldinger's honors and legacy

Honors 

Dr. Seldinger received numerous accolades for his contributions to medicine, including the Valentine Award from the New York Academy of Medicine in 1975 and the first Pioneer in Interventional Radiology Award from the Society of Interventional Radiology in 1992 [12]. He was also granted honorary memberships by the Swedish Association of Medical Radiology and the German Roentgen Association, recognizing his profound impact on the field.

Legacy in Medical Practice

Seldinger's legacy extends beyond his technique. He is remembered not only for his groundbreaking work but also for the way it transformed interventional radiology into a primary medical specialty. His approach paved the way for the development of numerous minimally invasive procedures that are now commonplace, such as angioplasty, embolization, and various catheter-based treatments [13].

Sven Ivar Seldinger's innovative technique has been lauded by several prominent figures in the field. Charles T. Dotter highlighted the monumental impact of Seldinger’s simple yet transformative method on reducing unnecessary surgical interventions and enhancing patient safety. Ronald G. Grainger commended it as a "medical milestone" that fundamentally changed radiologic practices, while Herbert L. Abrams praised its simplicity and effectiveness, crediting it for the advancements in diagnostic and interventional radiology that have greatly benefited patient care [14]​​. These testimonials collectively underscore Seldinger's enduring legacy and his pivotal role in shaping modern medicine.

Continued Influence

The "Seldinger Technique" remains a cornerstone in medical education and practice, and is taught extensively to medical students and professionals in the field of radiology and beyond. It exemplifies innovation that combines simplicity with profound utility, making it one of the most significant advances in medical practice during the 20th century [13,14]​.

## Conclusions

In summary, Sven Ivar Seldinger's introduction of a less invasive method for vascular access has not only enhanced the capability of medical practitioners to perform a wide range of procedures safely but has also greatly reduced patient risk and discomfort. His honors and enduring legacy reflect his pivotal role in transforming medical practices worldwide.

His great work gave physicians a new elegant path to the vessels of the body. Guidewires and needles all evolved through the years, but the idea of catheterization stayed the same. The percutaneous access of Seldinger's technique changed the way we see surgical treatment forever and paved the way for a new specialty: Interventional radiology. 
